# A Mitochondrion-Targeted Antioxidant Ameliorates Isoflurane-Induced Cognitive Deficits in Aging Mice

**DOI:** 10.1371/journal.pone.0138256

**Published:** 2015-09-17

**Authors:** Jing Wu, Huihui Li, Xiaoru Sun, Hui Zhang, Shuangying Hao, Muhuo Ji, Jianjun Yang, Kuanyu Li

**Affiliations:** 1 Jiangsu Key Laboratory of Molecular Medicine, Medical School of Nanjing University, Nanjing, China; 2 Department of Anesthesiology, Jinling Hospital, School of Medicine, Nanjing University, Nanjing, China; Virginia Tech University, UNITED STATES

## Abstract

Isoflurane possesses neurotoxicity and can induce cognitive deficits, particularly in aging mammals. Mitochondrial reactive oxygen species (mtROS) have been linked to the early pathogenesis of this disorder. However, the role of mtROS remains to be evaluated due to a lack of targeted method to treat mtROS. Here, we determined in aging mice the effects of the mitochondrion-targeted antioxidant SS-31, on cognitive deficits induced by isoflurane, a general inhalation anesthetic. We further investigated the possible mechanisms underlying the effects of SS-31 on hippocampal neuro-inflammation and apoptosis. The results showed that isoflurane induced hippocampus-dependent memory deficit, which was associated with mitochondrial dysfunction including reduced ATP contents, increased ROS levels, and mitochondrial swelling. Treatment with SS-31 significantly ameliorated isoflurane-induced cognitive deficits through the improvement of mitochondrial integrity and function. Mechanistically, SS-31 treatment suppressed pro-inflammatory responses by decreasing the levels of NF-κB, NLRP3, caspase 1, IL-1β, and TNF-α; and inhibited the apoptotic pathway by decreasing the Bax/Bcl-2 ratio, reducing the release of cytochrome C, and blocking the cleavage of caspase 3. Our results indicate that isoflurane-induced cognitive deficits may be attenuated by mitochondrion-targeted antioxidants, such as SS-31. Therefore, SS-31 may have therapeutic potentials in preventing injuries from oxidative stresses that contribute to anesthetic-induced neurotoxicity.

## Introduction

Postoperative cognitive dysfunction (POCD) tends to be a major health concern in elderly patients after surgery and anesthesia [1]. General anesthesia (GA)-induced neurotoxicity is increasingly emphasized as a key factor in the pathogenesis of POCD. Current hypotheses attribute direct neuro-inflammatory and apoptotic effects of anesthetics as the pathophysiological basis of GA-induced learning and memory impairments. The exact mechanisms by which GA-induced neurotoxicity are still not fully understood, and an effective pharmacologic treatment to rapidly reverse GA-induced cognitive deficits is lacking [2–6].

Mitochondrial reactive oxygen species (mtROS) have been linked to the earliest pathogenesis of isoflurane-induced cognitive deficits [7–9]. It is thought to initiate the pathogenesis of pro-inflammatory responses and apoptotic activation, which can subsequently lead to cognitive dysfunction in several neurodegenerative diseases [7–9]. Moreover, an increasing number of studies point to targeting mtROS as a novel therapy for inflammatory diseases and cancers, indicating that mitochondrion-targeted antioxidants can be powerful tools for investigating the role of mtROS in many processes both *in vitro* and *in vivo* [10–14]. However, little is known of the role of mitochondrion-targeted antioxidants in GA-induced neurotoxicity and their implications for cognitive function in aging brain.

The peptide Szeto-Schiller (SS)-31 (D-Arg-dimethylTyr-Lys-Phe-NH_2_) has an alternating aromatic-cationic structure that allows it to freely cross the blood-brain barrier and cell membrane, then concentrates >1000 fold in the mitochondrial inner membrane independently of mitochondrial membrane potential. It can scavenge various ROS and improve ATP production in many animal models [15–19]. These antioxidative effects are the basis of a new strategy to rapidly reverse cognitive deficits in GA-induced neurotoxicity, at least in mouse models.

In a model of isoflurane-induced cognitive deficits in aging mice, the hippocampus, an important tissue for learning and memory, is an affected area [20, 21]. We hypothesized that the activation of the mtROS-mediated inflammation and apoptosis is involved in the development of GA-induced cognitive deficits and that mitochondrion-targeted antioxidants might deactivate mtROS-mediated inflammation and apoptosis, and thus attenuate GA-induced cognitive deficits. In this study, we used a model of isoflurane-induced cognitive deficits in aging mice and SS-31, a mitochondrion-targeted antioxidant, to test our hypothesis.

## Materials and Methods

### Animals

Seventy-two fifteen-month-old male C57BL/6 mice were purchased from the Animal Center of Jinling Hospital, Nanjing, China. All experimental procedures and protocols were reviewed and approved by the Animal Investigation Ethics Committee of Jinling Hospital and were done in accordance with the Guidelines for the Care and Use of Laboratory Animals from the National Institutes of Health, USA. The mice were housed in a room maintained under constant environmental conditions with temperature 22–24°C and a 12-h light/dark cycle.

### Experimental protocols

Mice were randomly assigned to one of the following four groups (n = 18 each): control, control + SS-31, isoflurane, and isoflurane + SS-31. SS-31 (5 mg/kg, China Peptides Co, Ltd, Shanghai, China) or phosphate-buffered saline (PBS; vehicle) was intraperitoneally administered to the mice with a volume of 0.4 mL/kg 30 min before gas inhalation. The dose of SS-31 was chosen based on previous optimization in mouse models [15–18]. Anesthesia was induced by placing the mice in an anesthetizing chamber prefilled with 1.8% isoflurane plus 30% oxygen (O_2_) for 10 min and then changed to 1.5% isoflurane for 110 min. For control experiments, 30% O_2_ was delivered for 2 h at the same flow rate. The composition of the chamber gas was continuously monitored using a Datex^TM^ infrared analyzer (Capnomac, Helsinki, Finland). Mice were kept normothermic throughout the experiment.

Two experiments were performed. In experiment one, six of ten mice in each group were immediately decapitated 2 h after gas inhalation, and the hippocampus was rapidly removed and separated into two halves for mitochondria isolation and biochemical assays, respectively. The other four mice in each group were perfused for immunohistochemical analyses. In experiment two, eight mice in each group were used for behavioral tests 24 h after the 2 h-gas inhalation.

### Isolation of hippocampus mitochondria

Hippocampal tissues were homogenized in ice-chilled Dounce homogenizers (1:10, w/v) using isolation buffer (Beyotime Institute of Biotechnology, Shanghai, China) and centrifuged at 1,000 g for 5 min at 4°C. Supernatants were removed and centrifuged at 12,000 g to obtain pure cytosol fractions, which were isolated to determine cytosolic cytochrome C (Cyt C) levels. The mitochondria-enriched pellets were gently resuspended and washed with isolation buffer, then pelleted by centrifugation at 12,000 g for 5 min. Mitochondria were lysed and the protein was determined by the Micro BCA protein assay kit (Beyotime Institute of Biotechnology).

### Determination of mitochondrial swelling

The hippocampal mitochondrial swelling assay was performed by measuring the changes in the absorbance of the mitochondrial suspensions at 540 nm using a colorimetric assay kit (Genmed Scientifics Inc., Arlington, MA). A decrease in absorbance represents brain mitochondrial swelling.

### ROS and ATP content

Intracellular ROS was detected using a ROS assay kit (Genmed Scientifics Inc.) containing an oxidation-sensitive fluorescent probe (DCFH-DA) in a spectrofluorometer (excitation 490 nm, emission 520 nm). Assessment of relative ATP contents was performed using the ATP bioluminescence assay kit (Beyotime Institute of Biotechnology) following the manufacturer’s instructions.

### Western blotting analysis

The hippocampus from each animal was harvested and homogenized. Total protein (35 μg/lane) was electrophoretically separated and blotted onto nitrocellulose membranes. Protein levels were determined via incubation with antibodies against Inhibitory Nuclear Factor kappa (IκBα, 1:500; Santa Cruz Biotechnology), Bcl (1:500; Santa Cruz Biotechnology), Bax (1:500; Santa Cruz Biotechnology), Cyt C (1:5,000; Abcam), cleaved caspase 3 (1:1,000; Cell Signaling Technology), NLRP3 (1:500; Thermo Scientific), caspase 1 (cleaved p10, 1:200; Santa Cruz Biotechnology), voltage-dependent anion channel (VDAC, 1:1,000; Cell Signaling Technology), and β-actin (1:1,000; Cell Signaling Technology). Bands were visualized by enhanced chemiluminescence and quantified with the Image Quant Software (Syngene).

### Enzyme-linked immunosorbent assay (ELISA)

We detected the hippocampal levels of IL-1β and TNF-α at 24 h after isoflurane exposure by ELISA kits following the protocols provided by the manufacturer (Abcam). Readings were normalized to the amount of a standard protein.

### Immunohistochemical analysis

Animals (n = 4 for each group) were perfused with normal saline, followed by 4% paraformaldehyde 2 h after isoflurane or O_2_ exposure. Brain tissues were then immersed in 4% paraformaldehyde for later embedding. Paraffin sections were deparaffinized and hydrated using the following incubation steps: 10 min in xylene twice; 5 min in 100%, 10 min in 95%, 10 min in 85%, and 10 min in 70% ethanol; and 5 min three times in PBS at room temperature. Antigen retrieval was achieved by boiling the sections in 10 mM sodium citrate for 10 min in a microwave oven. The sections were washed with PBS three times, and treated with 3% H_2_O_2_-methanol for 15 min. Immunostaining was performed by incubation with antibodies against cleaved caspase 3 (1:200; Cell Signaling Technology), nuclear factor κB (NF-κB) p65 (1:100, Jiancheng Biological Technology Co. Ltd., Nanjing, China), Bax (1:100, Jiancheng Biological Technology Co. Ltd.) and Bcl-2 (1:100, Jiancheng Biological Technology Co. Ltd.) for 2 h. Sections were then washed three times and incubated with secondary antibodies labeled with horseradish peroxidase for 30 min at room temperature. Cells with brownish-yellow cytoplasm were counted as positive cells. The numbers of caspase 3, NF-κB, Bax and Bcl-2 immunoreactive cells in the hippocampal CA1 region were counted by an investigator blinded to the treatment conditions.

### TUNEL fluorescent assay

The TUNEL assay was carried out with the In Situ Cell Death Detection Kit (Roche Inc., Indianapolis, IN) following the protocols. Sections were counterstained by Anti-NeuN Antibody (1:200, Merck Millipore) for 3 min, washed with PBS three times, and covered by microscopic glass with Antifade Mounting Medium (Beyotime) for further analyses. TUNEL-positive cells in the hippocampal CA1 region were visualized and counted using a ZEISS HB050 inverted microscope system (Zeiss, Jena, Germany). The density of TUNEL positive cells in CA1 region was calculated by dividing the number of TUNEL-positive cells by the area of CA1 region.

### Open field test

To evaluate the anxiety behavior and general locomotor activity, mice were gently placed in the center of a white plastic chamber (40 cm × 40 cm × 40 cm) for 5 min, while exploratory behavior was automatically recorded by a video tracking system (XR-XZ301, Shanghai Soft maze Information Technology Co, Ltd., Shanghai, China). After each test, the arena was cleaned with 75% alcohol to avoid the presence of olfactory cues.

### Fear conditioning test

To measure the abilities of learning and memory, we employed the fear conditioning paradigm (30 cm long × 26 cm wide × 22 cm high, XR-XC404, Shanghai Softmaze Information Technology Co. Ltd.). Each mouse was exposed in the conditioning chamber for 3 min of accommodation then one tone-foot-shock pairing (tone, 30 s, 65 dB, 3 kHz; foot-shock, 3 s, 0.75 mA) was delivered. The contextual fear conditioning test was performed 24 h later by placing mice back to the same test chamber for 5 min without any stimulation. Two hours later, each mouse was placed in a novel chamber altered in shape, color, and smell, and the same tone was presented for 3 min without the foot shock to evaluate tone fear conditioning. Cognitive deficits in the test were assessed by measuring the length of time of “freezing behavior”, which is defined as a completely immobile posture except for respiratory efforts. Freezing behaviors were automatically recorded by the video tracking system.

### Statistical analysis

Data are presented as the mean ± SEM and analyzed by the Statistical Product for Social Sciences (SPSS; version 17.0, IL). The difference between groups was determined by one-way analysis of variance followed by the Bonferroni test. A *p* value <0.05 was regarded as statistically significant.

## Results

### SS-31 prevented ROS generation and improved ATP synthesis in the hippocampus in aging mice exposed to isoflurane

**Fig 1:**
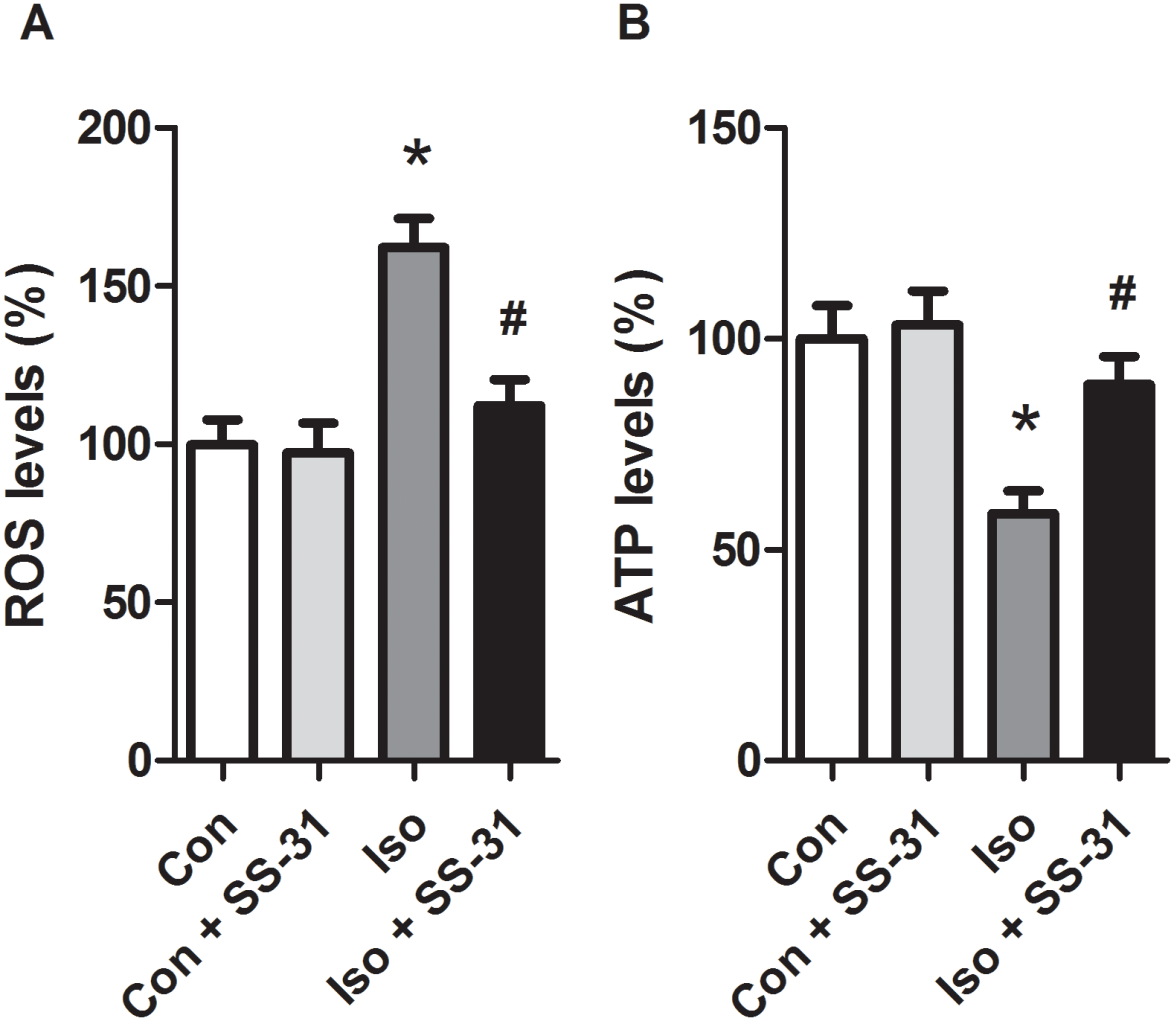
Effects of isoflurane on anesthesia and SS-31 pretreatment on ROS generation and ATP content in the hippocampus of aging mice. Peptide SS-31 (5 mg/kg) or PBS (vehicle) was intraperitoneally administered to 15-months old mice with a volume of 0.4 mL/kg 30 min before gas inhalation. Anesthesia was induced by placing the mice in an anesthetizing chamber prefilled with 1.8% isoflurane plus 30% oxygen for 10 min then changed to 1.5% isoflurane for 2 h. For control experiments, 30% O_2_ was delivered for 2 h at the same flow rate. Con: control group without any intervention; Con + SS-31: control mice treated with SS-31; Iso: mice treated with isoflurane; Iso + SS-31: mice treated with SS-31 and isoflurane. ROS levels (A) and ATP production (B) in the hippocampus were measured immediately after the samples were prepared (See Materials and Methods) in six mice from each group. Values are presented as mean ± SEM (n = 6). *p < 0.05 versus the control group; ^#^ *p* < 0.05 versus the isoflurane group.

Since isoflurane-induced cognitive deficits are accompanied with increased ROS levels and decreased ATP generation [21–23], we examined whether the mitochondrion-targeted antioxidant SS-31 could prevent ROS generation and improve ATP synthesis in the hippocampus after exposure to isoflurane in aging mice. The results confirmed that isoflurane induced a significant increase in ROS levels (Fig 1A) and a reduction in ATP contents in the hippocampus (Fig 1B). Moreover, the changes were completely reversed by SS-31 pretreatment (Fig 1A and 1B). These results indicate that SS-31 provides protective effects most likely through scavenging mitochondrial ROS and improving energetics in the hippocampus of aging mice exposed to isoflurane for 2 h.

### SS-31 suppressed the NLRP3 inflammasome-mediated cytokines in aging mice exposed to isoflurane

**Fig 2:**
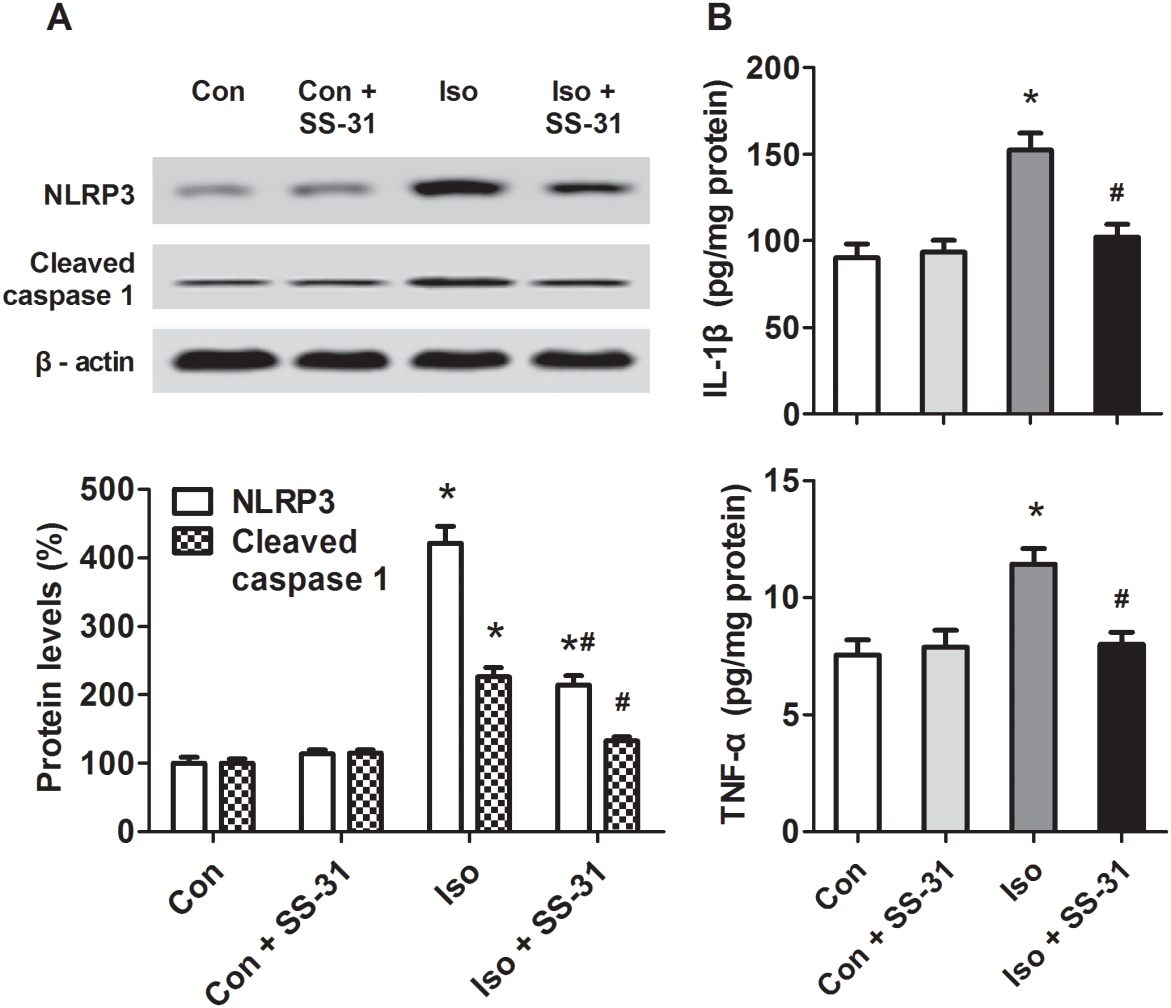
Effects of SS-31 pretreatment on the NLRP3 inflammasome-mediated inflammatory cytokines in the hippocampus of aging mice. (A) Western blotting analyses of NLRP3 and cleaved caspase 1 levels. (B) ELISA assays of IL-1β and TNF-α levels. Values are presented as mean ± SEM (n = 6). *p < 0.05 versus the control group; ^#^ *p* < 0.05 versus the isoflurane group.

An increasing body of evidence linking mtROS to inflammation suggests that mtROS act as signaling molecules to trigger pro-inflammatory cytokine production [24–27]. Here, we detected the protein levels of NLRP3, a marker of the inflammasome, which may further activate caspase 1. The downstream inflammatory cytokines IL-1β and TNF-α as critical pathophysiological cues of GA-induced neurotoxicity, were also determined. The results showed that NLRP3, cleaved caspase 1, IL-1β, and TNF-α (Fig 2A and 2B) were all upregulated 24 h after isoflurane exposure in the hippocampus of mice, but SS-31 pretreatment significantly suppressed the expression of NLRP3, cleaved caspase 1, and inflammatory cytokines (Fig 2A and 2B). Therefore, our results suggest that activation of NLRP3 inflammasome is involved in GA-induced neurotoxicity and mtROS are crucial to this activation.

### SS-31 suppressed mitochondrial apoptosis in aging mice exposed to isoflurane

**Fig 3:**
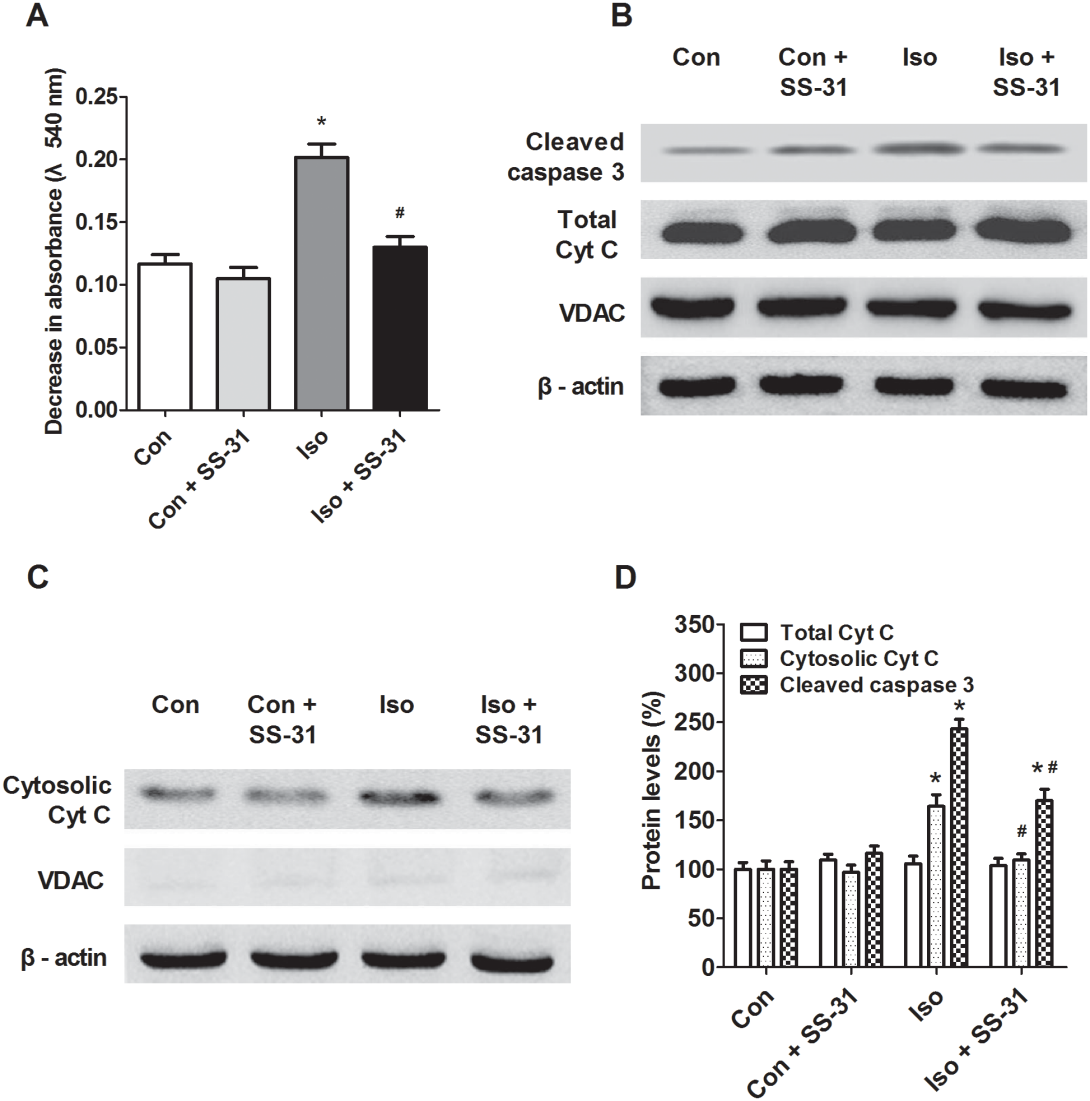
Effects of SS-31 pretreatment on the intrinsic mitochondrion-dependent apoptotic signaling pathway activity in the hippocampus of aging mice. (A) Swelling of mitochondria. (B) and (C) Protein levels of activated caspase 3, total Cyt C, and cytosolic Cyt C, revealed by western blotting analyses. Protein levels were normalized to VDAC for total protein and β-actin for the cytosolic fraction, respectively. Values are presented as mean ± SEM (n = 6). *p < 0.05 versus the control group; ^#^ *p* < 0.05 versus the isoflurane group.

**Fig 4:**
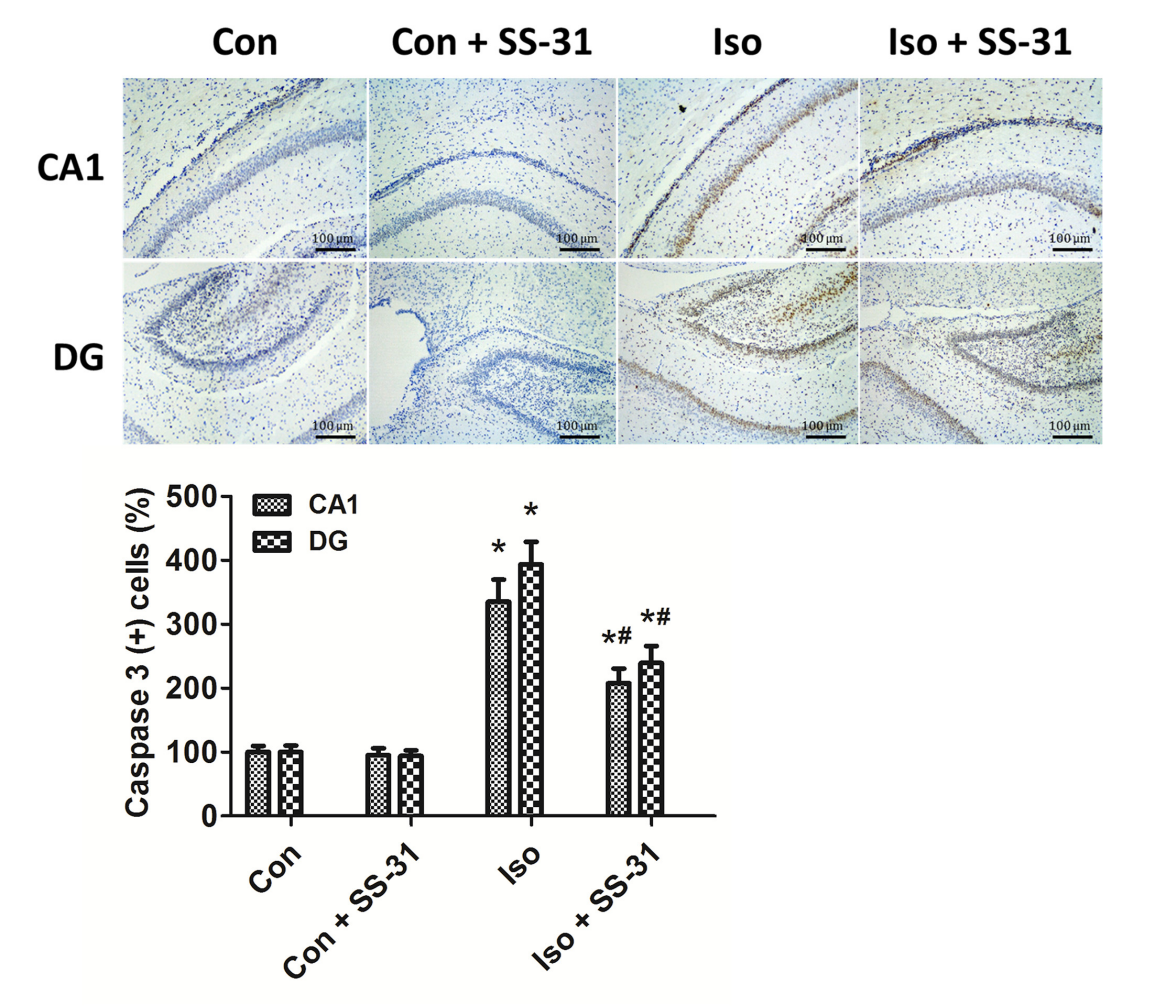
Activation of caspase 3 was attenuated by SS-31 pretreatment in mouse hippocampal CA1 and DG regions. Representative images of cleaved caspase 3 immunohistochemical (IHC) staining in the hippocampal CA1 and DG regions are shown. Cells with brownish-yellow cytoplasm are positive for cleaved caspase 3. Scale bar indicates 100 μm. Lower panel presents statistic data from the four experimental groups.

**Fig 5:**
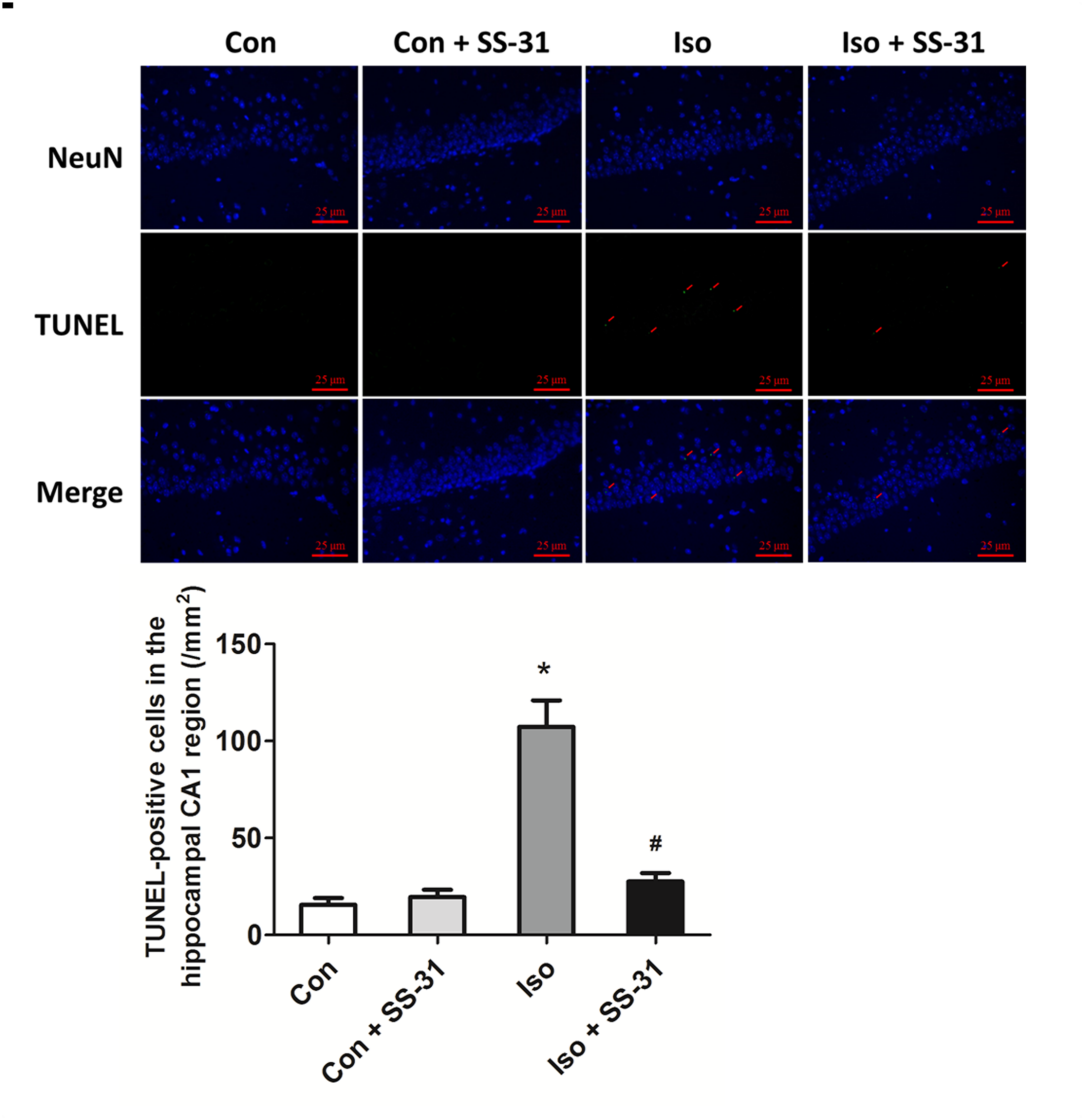
The number of TUNEL-positive cells was diminished by SS-31 pretreatment in mouse hippocampal CA1 regions. Representative images of TUNEL in the hippocampal CA1 region are shown. Green color indicates TUNEL-positive cells; blue, NeuN. Scale bar: 25 μm. The low panel shows statistical numbers of TUNEL-positive cells.

Isoflurane may induce ROS accumulation inside the mitochondria, leading to the opening of the mitochondrial permeability transition pore and releasing Cyt C to the cytosol, which initiates apoptosis through the mitochondrial pathway [4–6]. Consistently, our results showed that a 2h exposure to isoflurane induced an increase in mitochondrial permeability (Fig 3A), consequently leakage of Cyt C (Fig 3B, 3C and 3D), activation of caspase 3 (Figs 3B, 3D and 4), and apoptosis (Fig 5) in mouse hippocampus. However, SS-31 was protective against the intrinsic apoptosis in the hippocampus after anesthesia (Figs 3–5). These results support that isoflurane induces mitochondrion-dependent apoptosis and the mitochondrion-targeted peptide SS-31 presents therapeutic effects by inhibiting the activation of the apoptotic Cyt C-caspase 3 signaling pathway.

### SS-31 alleviated changes of IκBα, Bax, and Bcl-2 levels in the aging mice exposed to isoflurane

**Fig 6:**
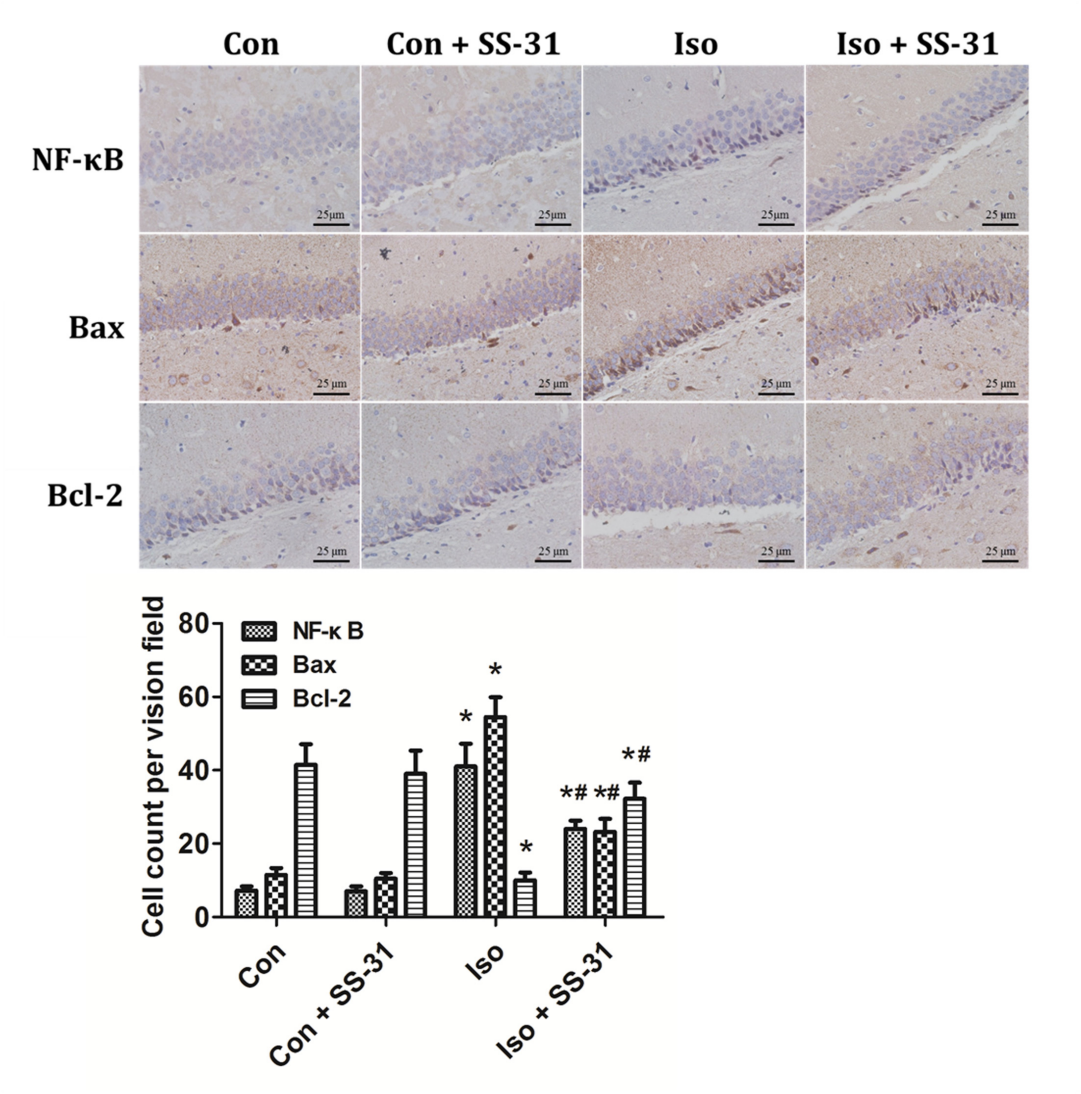
Effects of SS-31 pretreatment on cells positive for NF-κB p65, Bax, and Bcl-2 in the mouse hippocampal CA1 region. Representative images of NF-κB p65, Bax, and Bcl-2 cells immunohistochemical (IHC) staining in the hippocampal CA1 region are shown. Cells with brownish-yellow cytoplasm are positive for NF-κB p65, Bax, and Bcl-2. Scale bar: 25 μm. Lower panel represents statistic data from the four experimental groups.

**Fig 7:**
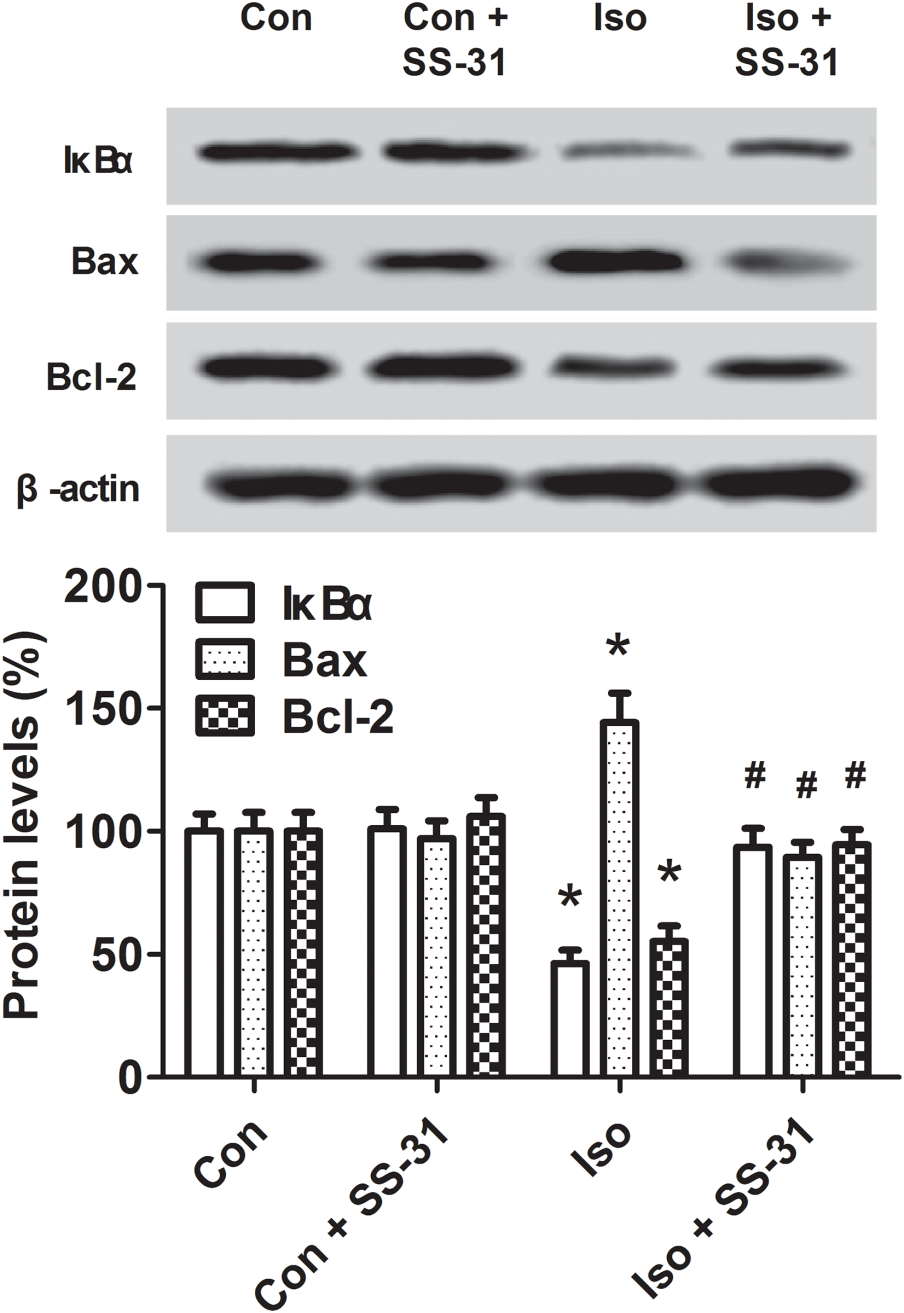
Effects of SS-31 pretreatment on IκBα, Bax, and Bcl-2 expression in the hippocampus of aging mice. Protein levels of IκBα, Bax, and Bcl-2 were revealed by western blotting analyses. Values are presented as mean ± SEM (n = 6). *p < 0.05 versus the control group; ^#^ *p* < 0.05 versus the isoflurane group.

To further investigate the role of mtROS and the effects of SS-31, we explored possible upstream signaling molecules in regulating inflammatory and apoptotic responses. One of the most important transcription factors, NF-κB, and two members of the Bcl-2 family, Bax and Bcl-2, were examined in the hippocampus of aging mice exposed to isoflurane. Western blotting analyses and immunohistochemical analyses revealed that SS-31 reduced the activation of NF-κB and Bax (Fig 6) and increased the expression of IκBα and Bcl-2 (Figs 6 and 7), which is consistent with a previous report [28]. Our results reveal that NF-κB and Bcl-2 family are associated with mtROS-induced neuroinflammation and apoptosis following anesthesia.

### SS-31 pretreatment ameliorates isoflurane-induced cognitive deficits in aging mice

**Fig 8:**
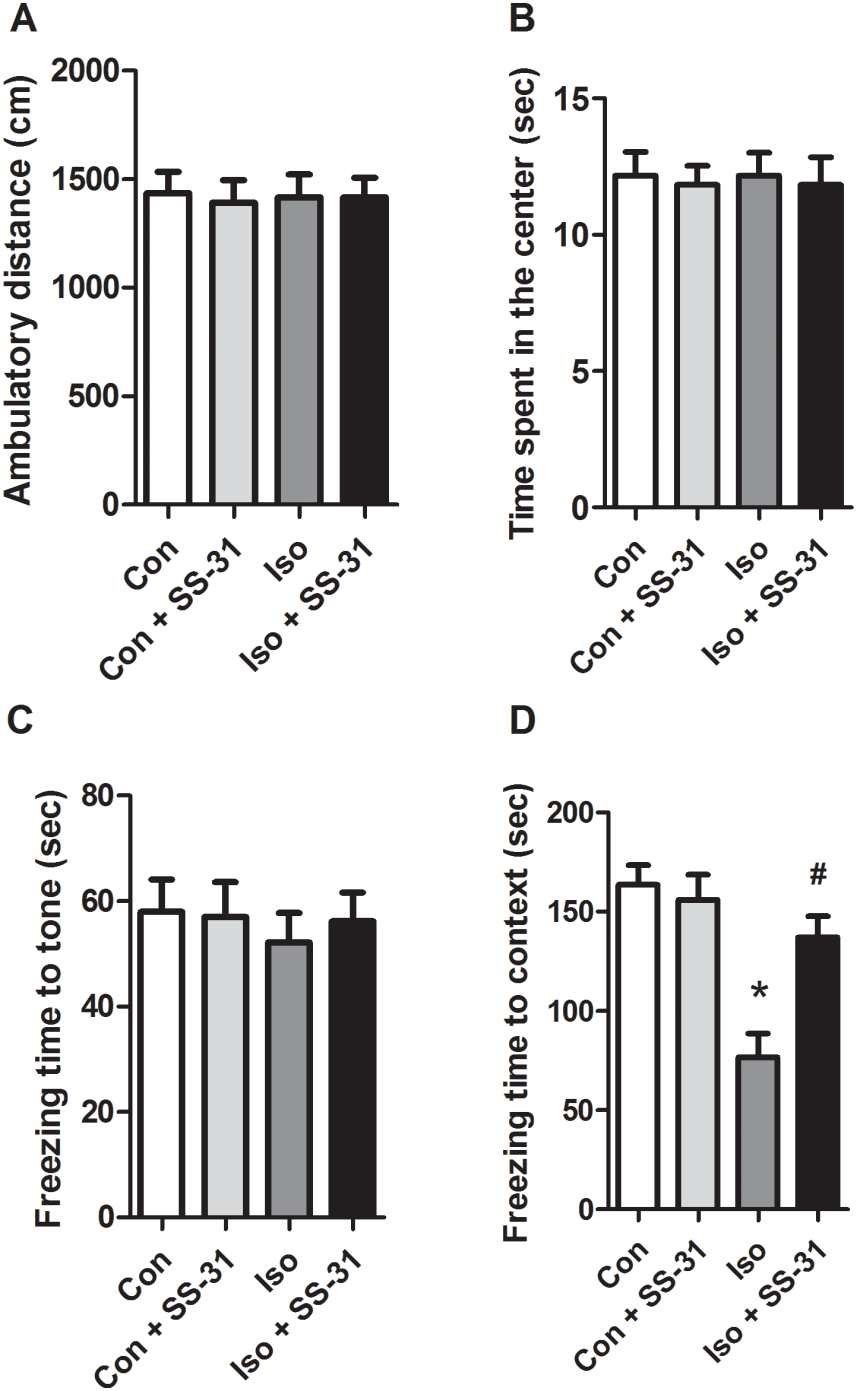
SS-31 pretreatment mitigated isoflurane-induced learning and memory impairments. Panels represent total distance traveled (A), time spent in the center (B), freezing time to tone (C), and freezing time to context (D), respectively. Values are presented as mean ± SEM (n = 8). *p < 0.05 versus the control group; *#p* < 0.05 versus the isoflurane group. The procedures of the open field test and fear conditioning test are in **Materials and Methods**.

To further verify the beneficial effects of SS-31 beyond histological improvement, open field and fear conditioning tests were performed to evaluate prevention of anesthesia-induced cognitive deficits 24 h after anesthesia. The values from these tests represent the general locomotor activity and abilities of the associated learning and memory. The results showed that the total distance traveled, time spent in the open field test, and freezing time in the 24 h-tone test were not different among the four groups (Fig 8A, 8B, and 8C). However, decreased freezing time in the 24 h-context test was observed in isoflurane-treated mice compared to that in the control mice (Fig 8D). Pretreatment with SS-31 reversed isoflurane-induced effects on freezing time (Fig 8D), reflecting an amelioration of hippocampus-dependent memory loss. These results reveal protective effects of SS-31 on isoflurane-induced cognitive deficits.

## Discussion

Here we show that, for the first time to our knowledge, SS-31 could ameliorate isoflurane-induced cognitive deficits in aging mice. The mechanism was that isoflurane induces the generation of mtROS, leading to the activation of NF-κB and upregulation of the pro-apoptotic factor Bax. The mitochondrion-targeted antioxidant SS-31 scavenges isoflurane-induced mtROS to prevent the occurrence of inflammation and apoptosis, protecting mice from hippocampus-dependent memory deficit.

Neuro-inflammation, more related to pro-inflammatory cytokine accumulation than microglia activation, has been proposed to be a possible pathogenic mechanism for GA-induced cognitive deficits [29, 30]. NLRP3 is the most widely studied inflammasome. It may activate caspase 1 and result in processing and secretion of the pro-inflammatory IL-1β and IL-18, which is implicated in several metabolic and inflammatory diseases [31–34]. In the present study, isoflurane consistently induced activation of NLRP3, cleaved caspase 1, IL-1β and TNF-αin the hippocampus of aging mice. Many factors are involved in the activation of the NLRP3 inflammasome. NF-κB is one of the most important transcription factors associated with inflammation, immune responses, cell survival, and proliferation. It is also reported as an essential signal that promotes the expression of NLRP3 and substrates IL-1β and IL-18 [35]. Here, our results support that NF-κB is associated with isoflurane-induced activation of NLRP3 inflammasome. Several lines of evidence have demonstrated that mtROS are essential for priming the activation of NF-κB and NLRP3 inflammasome [24, 36, 37]. Thus, it is rational that scavenging mtROS by SS-31 could suppress the increases of NF-κB, NLRP3, cleaved caspase 1, IL-1β, and TNF-α in aging mice after isoflurane exposure. To clarify that the activation of the NLRP3 inflammasome directly results from upregualted NF-κB and results in the activation of caspase 1, treatment of mice with NF-κB or caspase 1 inhibitors will be needed. Whether other canonical inflammasomes including NLRP1, NLRC4, and AIM2 are involved in the process remains unknown.

Activation of the intrinsic mitochondria-dependent apoptotic pathway appears to be the earliest sign of GA-induced developmental neuronal injury [38–40]. Overproduction of mtROS inhibits mitochondrial electron transport, which may result in mitochondrial membrane depolarization to initiate apoptosis by releasing Cyt C, as demonstrated in this study and previous work [41, 42]. Though recent views point to targeting mtROS as a novel therapy for GA-induced apoptosis, extensive studies are still lacking [43, 44]. Our study also provided evidence that the mitochondrion-targeted antioxidant SS-31 significantly inhibited apoptosis and prevented cognitive deficits in GA-treated mice. Thus, removal of mtROS protects mitochondrial integrity and function from damage and further reduces hippocampus-dependent memory defects in aging mice. These results indicate that mtROS play an important role after isoflurane exposure and that SS-31 might be considered as a promising agent for the treatment of POCD.

In conclusion, our results suggest that isoflurane-induced cognitive deficits are associated with generation of mtROS, which activates the NLRP3 inflammasome and the apoptotic Cyt C-caspase 3 signaling pathway. The mitochondrion-targeted antioxidant SS-31 may attenuate isoflurane-induced loss of hippocampus-dependent memory in aging mice. The therapeutic implication points to mtROS as a potent target for GA-induced cognitive deficits.
